# Comparative analysis of mitochondrial genomes of *Broussonetia* spp. (Moraceae) reveals heterogeneity in structure, synteny, intercellular gene transfer, and RNA editing

**DOI:** 10.3389/fpls.2022.1052151

**Published:** 2022-12-01

**Authors:** Chanjuan Lai, Jie Wang, Shenglong Kan, Shuo Zhang, Pan Li, Wayne Gerald Reeve, Zhiqiang Wu, Yonghua Zhang

**Affiliations:** ^1^ College of Life and Environmental Science, Wenzhou University, Wenzhou, China; ^2^ Shenzhen Branch, Guangdong Laboratory for Lingnan Modern Agriculture, Genome Analysis Laboratory of the Ministry of Agriculture, Agricultural Genomics Institute at Shenzhen, Chinese Academy of Agricultural Sciences, Shenzhen, China; ^3^ Kunpeng Institute of Modern Agriculture at Foshan, Foshan, Guangdong, China; ^4^ College of Science, Health, Engineering and Education, Murdoch University, Western Australia, Australia; ^5^ Laboratory of Systematic & Evolutionary Botany and Biodiversity, College of Life Sciences, Zhejiang University, Hangzhou, Zhejiang, China

**Keywords:** paper mulberry, organellar genome, genomic configuration, synteny, sequence migration, post-transcribed editing

## Abstract

The genus *Broussonetia* (Moraceae) is comprised of three non-hybrid recognized species that all produce high quality fiber essential in the development of papermaking and barkcloth-making technology. In addition, these species also have medicinal value in several countries. Despite their important economical, medicinal, and ecological values, the complete mitogenome of *Broussonetia* has not been reported and investigated, which would greatly facilitate molecular phylogenetics, species identification and understanding evolutionary processes. Here, we assembled the first-reported three complete *Broussonetia* (*B. papyrifera*, *B. kaempferi*, and *B. monoica*) mitochondrial genomes (mitogenome) based on a hybrid strategy using Illumina and Oxford Nanopore Technology sequencing data, and performed comprehensive comparisons in terms of their structure, gene content, synteny, intercellular gene transfer, phylogeny, and RNA editing. Our results showed their huge heterogeneities among the three species. Interestingly, the mitogenomes of *B. monoica* and *B. papyrifera* consisted of a single circular structure, whereas the *B. kaempferi* mitogenome was unique and consisted of a double circular structure. Gene content was consistent except for a few transfer RNA (tRNA) genes. The *Broussonetia* spp. mitogenomes had high sequence conservation but *B. monoica* with *B. kaempferi* contained more synteny blocks and were more related, a finding that was well-supported in organellar phylogeny. Fragments that had been transferred between mitogenomes were detected at plastome hotspots that had integrated under potential mediation of tRNA genes. In addition, RNA editing sites showed great differences in abundance, type, location and efficiency among species and tissues. The availability of these complete gap-free mitogenomes of *Broussonetia* spp. will provide a valuable genetic resource for evolutionary research and understanding the communications between the two organelle genomes.

## Introduction

*Broussonetia* belongs to the family Moraceae, the nitrogen-fixing clade in the APG IV and contains the non-hybrid species *B. monoica*, *B. kaempferi*, *B. papyrifera*, and one hybrid species (*Broussonetia* × *kazinoki*). These species can be found across East Asia (China, Korea, Japan), Southeast Asia (Vietnam, Laos, Thailand, Cambodia, Myanmar), India, island Southeast Asia, Melanesia, and the Polynesia islands (Chang et al., 2015; Chung et al., 2017). *Broussonetia papyrifera* was essential in the development of the papermaking technology in ancient China and barkcloth-making in the Pacific Islands and Central America (Barker, 2002; Chang et al., 2015; Peng et al., 2019). Due to its important economical, medicinal, ecological attributes, *B. papyrifera* is being developed into a novel model system for woody plant research (Peng and Shen, 2018; Peng et al., 2019). The species *Broussonetia monoica* is a shrub (2–4 m tall) which exhibits notable leaf morphological diversity (Chung et al., 2017; Kuo et al., 2022), and *Broussonetia kaempferi* is a scandent shrub with highly suitable bark fibers that are used for making paper (Zhou and Michael, 2003). Recently, several studies have characterized the genus *Broussonetia* by molecular phylogeny (Chung et al., 2017), provided complete chloroplast genome assembly (Kuo et al., 2022) and an in-depth investigation into the chromosomal-scale genome of *B. papyrifera* (Peng et al., 2019). However, the dynamic evolution of mitochondrial gene and intron content of *Broussonetia* has never been assessed until now.

It has been greatly accepted that plant organellar genomes originate from ancient endosymbiotic bacteria approximately a billion years ago and play vital roles in massive essential life activities including photosynthesis, cellular respiration, and ATP synthesis (Woloszynska, 2009; Wu et al., 2022). The plastid genome (plastome)is a circular molecule, highly conserved in structure and gene content across most lineages, making it ideal for phylogeny (Wu and Ge, 2011; Wang et al., 2022). The mitochondrial genome (mitogenome) is semiautonomous encoding some self-related proteins but is still under co-regulation of products from nuclear-encoded genes (Palmer and Herbon, 1988; Burki, 2016). Mitogenomes of most land plants typically contain 40 to 60 genes that are inherited from a single parent (Palmer and Herbon, 1988). Compared to plastome, the mitogenome is usually larger in molecular weight. It varies widely in size, ranging from 208 kb (*Brassica juncea*) (Knoop, 2004) to over 2400 kb (*Cucumis melo*) (Ward et al., 1981), then to the largest known (*Larix sibirica*) of nearly 12 Mb (Putintseva et al., 2020). This is mainly due to the redundancy (multiple copies) of mitochondrial protein coding genes (PCGs) and the frequent recombination and integration of foreign DNA in the mitogenome (Mackenzie and McIntosh, 1999). Gene order, genome structure, and mitogenome size are highly variable in plants (Sloan et al., 2012a). The “evolutionary paradox” is an important evolutionary feature of plant mitogenome, that is, the mutation rate of plant mitogenome sequence is very low, but the rate of mitogenome structure rearrangement is really high (Palmer and Stein, 1986; Wolfe et al., 1987). The rate of nucleotide synonymous substitution in a plant mitogenome is several to dozens of times lower than that of the plastome and nuclear genome, and even 50 to 100 times lower than that of the mammalian mitogenome (Wolfe et al., 1987). Structural complexity is another important feature of plant mitogenomes. Physical mapping and sequencing of mitochondria in some species suggest that the complex structure of mitochondria is shaped by self-recombinant gene transfer to the nucleus and other unclear factors (Sloan, 2013). Structural analyses reveal frequent intramolecular and intermolecular recombination, resulting in a structurally dynamic assemblage of genome configurations, which makes the mitogenome a powerful model for studying genome structure and dynamic evolutionary patterns (Drouin et al., 2008). In addition, gene transfer is another kind of common event in plant mitogenomes which provides an essential contribution to the complexity of mitogenome. Intracellular gene transfer (IGT) is generally known as the mutual transfer of DNA between the two organelle genomes and the nuclear genome within the cell (Timmis et al., 2004). The mitogenome size variation largely reflects differences in the amount of non-coding content, which comes from diverse sources including repeats and large duplications (Alverson et al., 2011), IGT of nuclear and plastid DNA and horizontal gene transfer (HGT) from other species (Park et al., 2015).

In this study, we decoded the mitogenomes of three *Broussonetia* spp. (*B. monoica*, *B. kaempferi*, and *B. papyrifera*) based on a strategy of combining next-generation and third-generation sequencing methods. We comprehensively compared their genomic characteristics in terms of gene content, intracellular sequence transfer, mitogenomic synteny, organellar phylogeny and RNA editing sites, to investigate the intrageneric heterogeneity in terms of this maternally inherited organelle. These established mitogenomes will provide a valuable resource for future evolutionary and functional research on these traditional valuable taxa.

## Results

### Genome assembly and characterization

**Figure 1:**
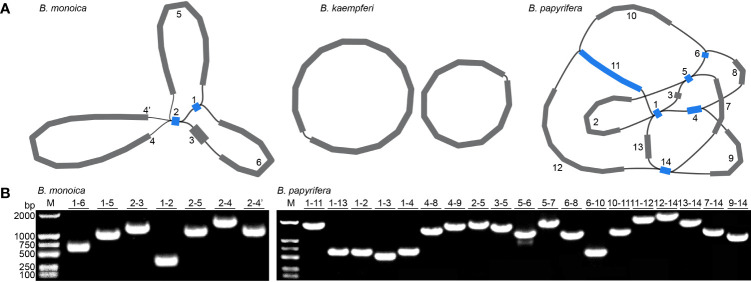
Mitogenome assembly graph and possible connections (black lines) mediated by repeats. **(A)** Mitogenome assembly graph of *B monoica*, *B kaempferi*, and *B papyrifera*. Unique and repeated contigs are colored in grey and blue, respectively. **(B)** PCR verification of possible connections.

**Figure 2:**
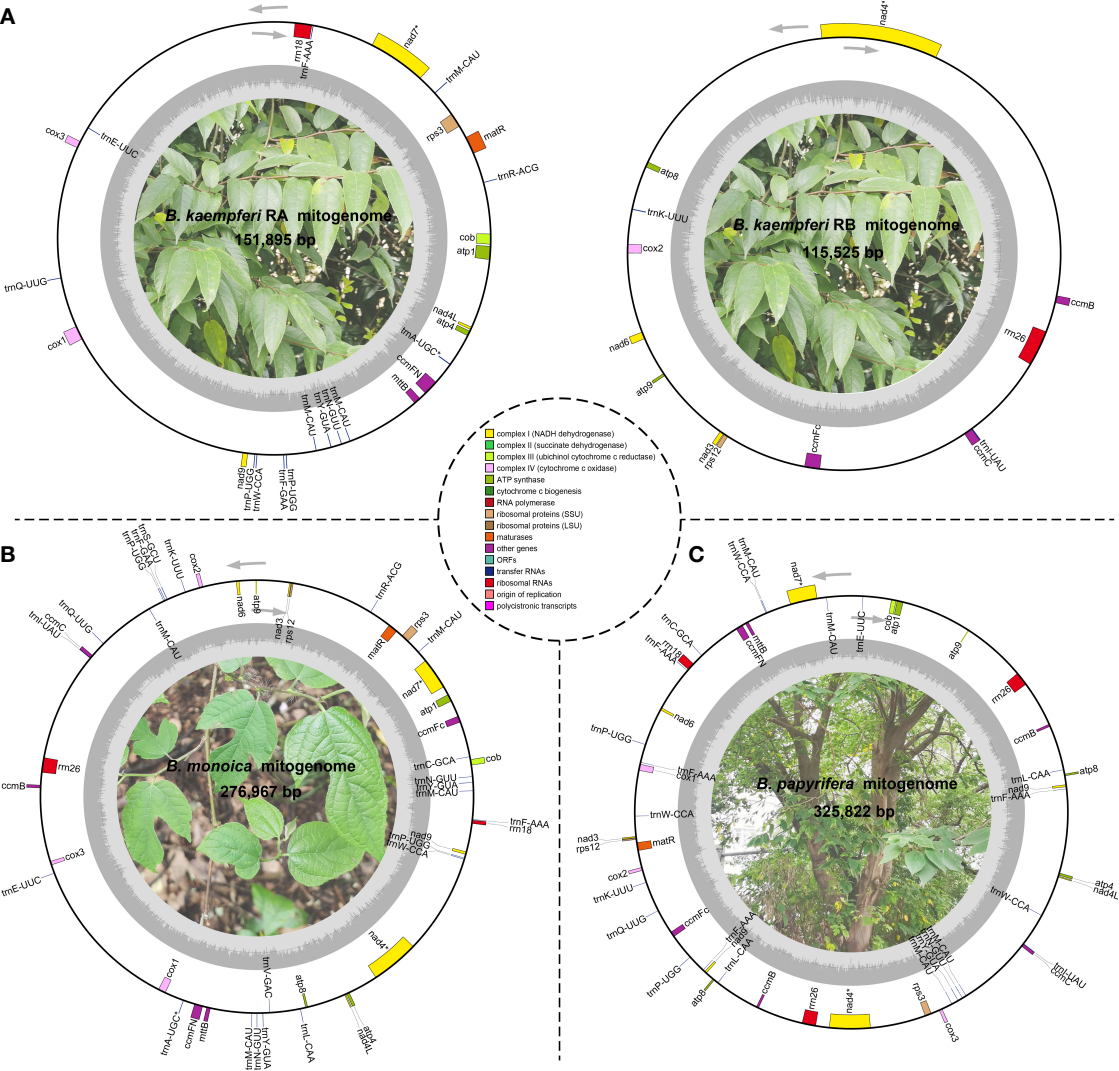
Schematic mitogenome diagram of three *Broussonetia* spp. in this study. **(A)** Two mitochondrial subrings (RA and RB) of *B kaempferi*. **(B)** *B monoica*. **(C)** *B papyrifera*. Genes belonging to different functional groups are color-coded. ^*^ indicates gene containing intron(s). *nad2* and *nad5* were trans splicing so they were not shown here.

Three accurate *Broussonetia* mitogenomes were obtained by combining Illumina and ONT reads. Consistent depths of mapping reads revealed the high quality gap-free assembly (**Figure S1**). A total of seven and 19 different possible connections mediated by two and six repeats in *B. monoica* and *B. papyrifera* mitogenomes, respectively, were generated by *de novo* assembly, of which all were verified by PCR (**Figure 1**; **Table S1**). A single-circular molecule was resolved in both *B. monoica* and *B. papyrifera* mitogenomes of size 276,967 bp with 5.43% GC content and 325,822 bp with 45.96% GC content respectively (**Figures 2B, C**). However, the mitogenome of *B. kaempferi* was a “double ring” form, with the coexistence of two independent molecules. The bigger ring (referred to as RA) was 151,895 bp in size with 45.80% GC content, while the smaller one (referred to as RB) was 115,525 bp in size and contained 45.16% GC content (**Figure 2A**). Therefore, the total size of the *B. kaempferi* mitogenome reached 267,420 bp. A total of 41, 39, and 37 unique mitochondrial genes were located in *B. monoica*, *B. kaempferi*, and *B. papyrifera*, and the three species shared 36 genes including all 23 protein-coding genes (PCGs), two rRNA genes, and 11 tRNA genes, while *trnA-UGC*, *trnC-GCA*, *trnL-CAA*, *trnR-ACG*, and *trnV-GAC* were absent from the mitogenome of *B. kaempferi* and/or *B. papyrifera* mitogenome (**Table 1**). In contrast to all PCGs which were single-copy, certain *rrn* or *trn* genes existed in multiple copy. The genes *rrn26* and *trnL-CAA* were uniquely duplicated in *B. papyrifera*, in addition *tRNF-AAA* had four copies and *trnW-CCA* had three copies in *B. papyrifera*. *trnN-GUU* and *trnY-GUA* were uniquely duplicated in *B. monoica*. In addition, *trnM-CAU* had four copies in both *B. monoica* and *B. papyrifera* but three copies in *B. kaempferi*. *trnP-UGG* gene was duplicated in both *B. monoica* and *B. papyrifera* (**Table 1** and **Figure 2**). A total of 112, 107, and 130 SSRs were detected in *B. monoica*, *B. kaempferi* (59 in RA and 48 in RB), and *B. papyrifera*, respectively. SSR mono-A and mono-T were the most abundant SSRs type, and tetra-nucleotide SSRs were also identified which varied in composition (**Figure S2**). Dispersed repeats across the mitogenomes were identified as two types, forward and palindromic matches. There were 91, 40, and 177 dispersed repeats in *B. monoica*, *B. kaempferi* (24 in RA and 16 in RB), and *B. papyrifera*, which accounted for 3.61% (10,003 bp), 1.90% (5075 bp), and 23.62% (76,968 bp) of the entire mitogenome. It was noticed that no transferred fragments with 301–500 bp were detected (**Figure S3**). Obviously high heterogeneity in structural conformation, gene copy counts, and repeated sequences indicated the massive complexity of mitogenome. Additionally, a total of 25 shared codons encoding 16 amino acids (one was a stop codon, UGA) showed that RSCU excessed 1 across all three *Broussonetia* spp. (**Table S2**), together with fixed PCG content indicated the high conservation in functional components.

**Table 1:** Known functional genes in the three *Broussonetia* spp. mitogenomes.

Product group	Gene
Complex I	*nad2, nad3, nad4, nad4L, nad6, nad7, nad9*
Complex III	*cob*
Complex IV	*cox1, cox2, cox3*
Complex V	*atp1, atp4, atp8, atp9*
Cytochrome *c* biogenesis	*ccmB, ccmC, ccmFc, ccmFN*
Ribosome	*rps12, rps3*
Other proteins	*matR, mttB*
rRNA	*rrn18, rrn26*
tRNA	*trnA-UGC* ^2^ *, trnC-GCA* ^2^ *, trnE-UUC, trnF-AAA, trnF-GAA, trnI-UAU, trnK-UUU, trnL-CAA^1^, trnM-CAU, trnN-GUU, trnP-UGG, trnQ-UUG, trnR-ACG* ^2^ *, trnV-GAC* ^3^ *, trnW-CCA, trnY-GUA*

^1^tRNA lost in B. kaempferi; ^2^ tRNA lost in B. papyrifera; ^3^ tRNA lost in both B. kaempferi and B. papyrifera.

**Figure 3:**
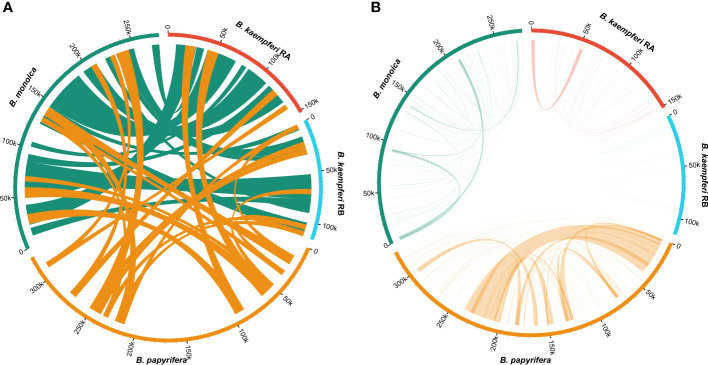
Synteny of the mitogenomes. **(A)** Interspecific synteny indicated by homologous regions longer than 5000 bp. **(B)** Individual internal synteny indicated by all identified homologous regions.

Synteny of the entire mitogenome among three *Broussonetia* spp. revealed massive interspecies large homologous regions experiencing structural recombination as numerous crosses could be observed (**Figure 3A**). In addition, small homologous regions could be found across the entire mitogenome, which revealed high compositional and low structural conservation (**Figure S4**). More homologous regions could be found between *B. monoica* and *B. kaempferi* than either with *B. papyrifera*, suggesting a closer relationship between *B. monoica* and *B. kaempferi*. When it came to individual internal synteny, there were obvious differences. There were fewer homologous regions that could be detected and most of them were very short except for a 33k-bp repeat in *B. papyrifera* (**Figure 3B**).

### IGTs between organellar genomes

**Figure 4:**
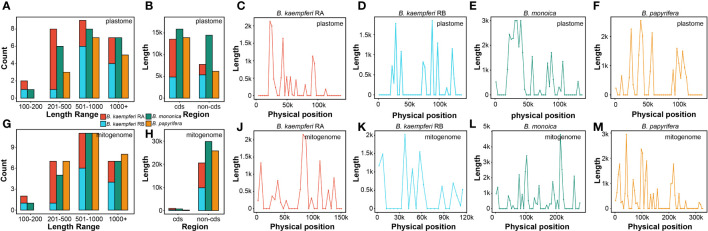
Transferred fragments from plastome to mitogenome across the three *Broussonetia* spp. in this study. **(A)** Length distribution of transferred fragments in plastome. **(B)** Type of transferred fragments in plastome. **(C)** Length distribution of transferred fragments in mitogenome. **(D)** Type of transferred fragments in mitogenome. **(E–H)** Location of transferred fragments across plastome. **(J–M)** Location of transferred fragments across mitogenome.

The global alignment between the two organellar genomes illustrated an uneven distribution of the homologous regions across some plastomic regions (**Figure S5**). A total of 22 (30,374 bp), 26 (21,308 bp), and 15 (20,085 bp) fragments were transferred from the plastome of *B. monoica*, *B. kaempferi*, and *B. papyrifera*, respectively, and were integrated into 24 (30,604 bp), 27 (21,545 bp), and 26 (26,037 bp) fragments in corresponding mitogenomes due to the active recombination and rearrangement. Length of transferred fragments in the plastome ranged from 174 bp in *B. kaempferi* RB to 9105 bp in *B. monoica*, while in the mitogenome it ranged from 174 bp in *B. kaempferi* RB to 7339 bp in *B. monoica*. The majority of transferred fragments for each species occurred was in the size range of 501–1000 bp either for either the plastome or mitogenome, but transferred fragments were observed from 100–200 bp and up to 1000+ bp (**Figures 4A, C**). It was noticed that although coding sequences (cds) transferred more frequently than non-cds across all three plastomes, non-coding regions were dominant in the integration locations across mitogenomes (**Figures 4B, D**). Across the plastome, transfer tended to happen in certain regions that appeared to be hotspots for rearrangement (**Figures 4E–H**), but there seemed no similar hotspots across the mitogenomes (**Figures 4J–M**). To investigate whether there was a particular pattern, the hotspots (peaking windows) that contained more than 2000 bp fragments were selected to investigate their content. There were six, four, and one hotspot in the plastome of *B. monoica*, *B. kaempferi*, and *B. papyrifera*, and the transferred fragments were relatively identical, including partial *psbZ-trnG-GCC* to partial *psaB* at 39k–43k and partial *rpoC1* to partial *rpoB* at 24k–27k for both *B. monoica* and *B. papyrifera*, and partial *rpoC2* to partial *rpoC1* at 21k–24k for both *B. monoica* and *B. kaempferi* (**Table S3**). For the peaking windows, five out of eleven windows contained at least one complete or partial tRNA. A total of six, three, and three hotspots across the mitogenomes of *B. monoica*, *B. kaempferi*, and *B. papyrifera*, respectively, were identified without any identical components. However, nine out of 12 of the windows contained at least one complete or partial tRNA (**Table S4**).

## Organellar phylogeny

**Figure 5:**
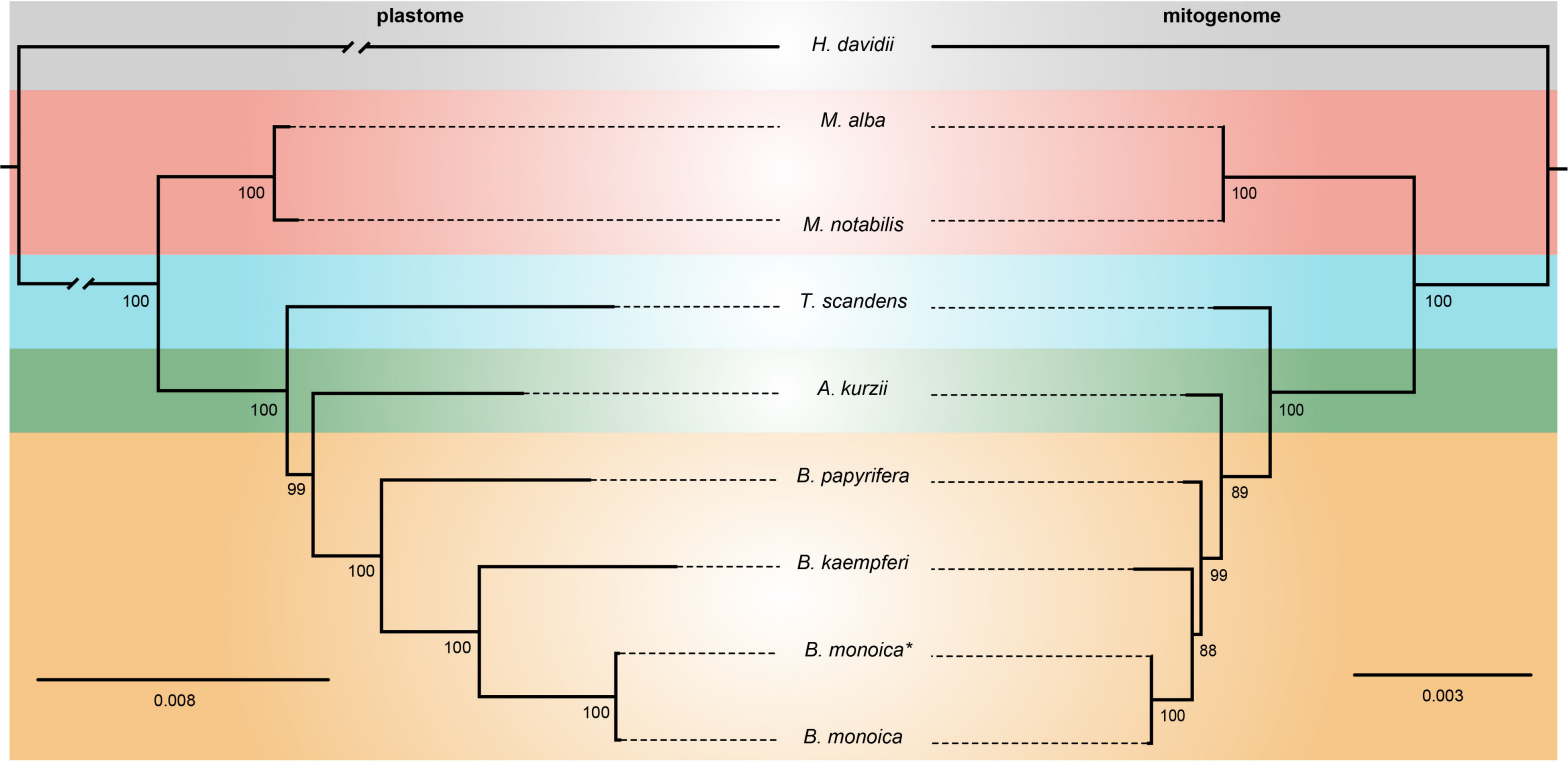
ML tree based on 73 shared plastid genes (left) and 13 shared mitochondrial genes (right). The numbers near nodes indicated the bootstrap value. *B monoica* ^*^ was newly sequenced in this study while *B monoica* was previously sequenced.

The plastome matrix was 54,143 bp in alignment size with 1200 sites with alignment gaps or missing data. A total of 4584 (8.47%) sites were variable, of which 3161 were singleton variable (3073 two-variants, 87 three-variants, and 1 four-variants) and 1423 were parsimony informative (1285 two-variants and 138 three-variants) (**Table S5**). The mitogenome matrix was 13,251 bp in total with 388 sites with alignment gaps or missing data, 276 (2.08%) sites were variable which consisted of 197 singleton variable (196 two-variants and 1 three-variants) and 79 parsimony informative sites (75 two-variants and 4 three-variants) (**Table S5**). The phylogenetic relationships revealed by plastome and mitogenome were identical here. In the plastome tree, all nodes were fully supported with the sole exceptional node supported by BS = 99, while in the mitogenome tree, there were two nodes with BS < 90. However, this would not influence greatly the definite phylogenetic relationship. *Broussonetia* was monophyletic with *Allaeanthus* as its sister. Within *Broussonetia*, *B. papyrifera* was well-supported to be sister to the *B. kaempferi* and *B. monoica* clade. Much shorter branch lengths in the mitogenome tree compared with the plastome tree indicated slower evolution of the mitogenome (**Figure 5**).

## RNA editing and NUMT transcription

**Figure 6:**
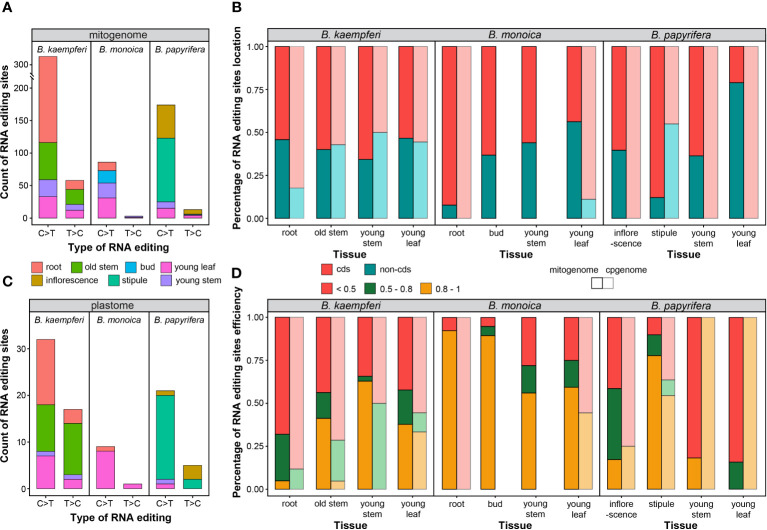
RNA editing type and efficiency. **(A)** Count of RNA editing site across mitogenome in each tissue. **(B)** Percentage of RNA editing site location in each tissue. **(C)** Count of RNA editing site across plastome in each tissue. **(D)** Percentage of RNA editing efficiency in each tissue.

The tissue-specific RNA editing site abundance and efficiency were compared in the *Broussonetia* spp. mitogenome and plastome. The mitogenome contained more than times of RNA editing sites than plastome did. Among interspecies, *B. kaempferi* had the richest (385, 104 in RA, and 281 in RB) while *B. monoica* contained the least (89) abundance, in terms of either type in mitogenome or plastome. The C>T was predominate accounting for 85to 97% in mitogenome and 65.3to 90.0% in plastome among the three species (**Figure 6A**). When it came to tissues within individuals, in *B. kaempferi*, mitochondrial sites were much more abundant in root (225, 58.4%) and plastid sites were more abundant in old stem (21, 42.9%). In the *B. monoica* mitogenome, there was no massive heterogeneity among the four tissues. But for the plastome, almost no tissues other than young leaf contained RNA editing sites. In *B. papyrifera*, the stipule possessed most sites in the both mitogenome (99, 52.9%) and the plastome (17, 73.9%) while inflorescence contained 58 (31.0%) in the mitogenome but only one (4.1%) in the plastome (**Figure 6C**). Interestingly, young leaf and young stem contained similar abundance in *B. kaempferi* and *B. monoica*, much more than those in *B. papyrifera* in each organelle genome (**Figures 6A, C**). RNA editing events tended to occur in different regions among three species (**Figure 6B**). In *B. kaempferi*, slight differences in location were observed in the mitogenome among the four tissues. In root and young stem, more sites were detected in cds across the plastome and the mitogenome, respectively. In *B. monoica*, huge differences could be found among tissues and organelle genomes. In root, more sites were located in cds showing a great gap with non-cds of the mitogenome while in the other three tissues, the gaps were not so huge, but in young leaf, the situation was reverse in the plastome. In *B. papyrifera*, more sites were in cds of mitogenome to various degrees except for young leaf with more sites in non-cds. RNA editing efficiency varied among species and tissues as well (**Figure 6D**). In *B. monoica*, high efficiency was shared in the mitogenome among four tissues especially in root and bud. In *B. kaempferi* mitogenome, only young stem contained over 50% sites with 0.8–1 efficiency as the stipule did in *B. papyrifera* mitogenome.

**Figure 7:**
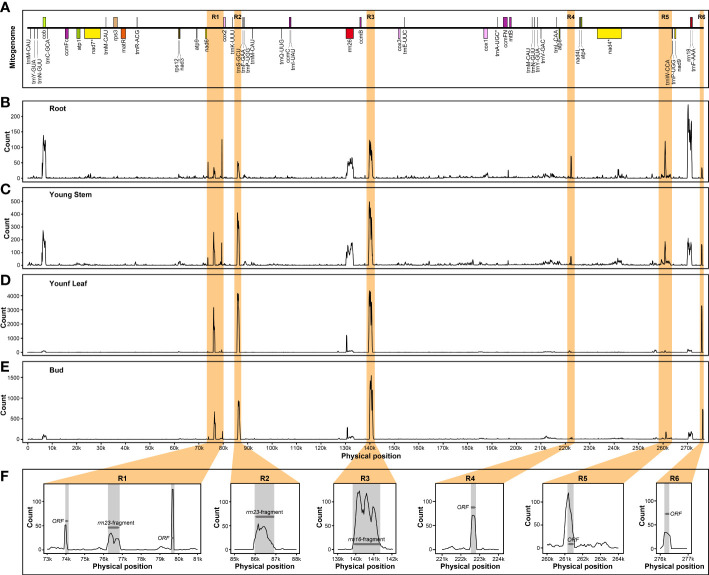
Transcript abundance of *B monoica* mitogenome transfer into nuclear genome in different tissues. R1 to R6 (orange regions) indicates six IGS with relatively high transcript abundance. **(A)** Liner annotation of *B monoica* mitogenome. **(B–E)** Count of RNA reads mapping to *B monoica* mitogenome in root, young stem, young leaf, and bud, respectively, using a 100-bp sliding window with a 50-bp step size. **(F)** Annotation of R1–R6. Grey regions indicate annotated gene fragments or open reading frames (ORFs) and arrows indicate the transcriptional direction.

In addition, several nuclear mitochondrial DNA segments (NUMTs) could be actively transcribed as components of nuclear genome regardless of different tissues. In *B. monoica*, there were several PCGs and six intergenic spacers (IGS) participating in the expression process, of which two were annotated as the residue of *rrn23* and *rrn26*, and four were open reading frames (**Figure 7**). Similarly, five transcribed NUMTs from IGS of the mitogenome were detected in *B. papyrifera*, and three and two were in *B. kaempferi* RA and RB, respectively (**Figures S6–S8**).

## Discussion

Plant mitogenomes have evolved from with dramatic and rapid structural changes, thus many unique features can be found compared with conserved plant plastomes and compact animal mitogenomes. These features include huge genome size variations, active multipartite arrangements, low gene density, abundant post-transcribed RNA editing, gene sequence transfer or loss, and foreign sequence capture (Hong et al., 2021; Wu et al., 2022). Although plant mitogenomes are conventionally reported as circular structures, much like the circular chromosomes found in animal mitochondria and bacteria, many studies have shown that the simple circular model of genome structure that applies to most animal species is inadequate when trying to understand plant mitochondria that can have multiple circular replicons, branched, linear, or mixed forms of genomic structure, i.e., *Silene noctiflora* has a mitogenome that is arranged into numerous circular chromosomes, that is, one ring cannot rule them all (Sloan, 2013; Wu et al., 2015) based on mapping to a circular reference. Here in this study, we obtained the first complete record of *Broussonetia* mitogenomes by providing three gap-free assemblies based on a hybrid strategy combining Illumina and ONT long sequencing reads. During *de novo* assembly, heterogeneity could be readily founded in configuration mediated by repeats that have potential to mediate structural transformation as all the connections were verified by PCR experimental methods (**Figure 1**). It also showed that the three *Broussonetia* mitogenomes showed wide differences in structure. In particular, such a configuration consisting of double molecules was observed in the *B. kaempferi* mitogenome rather than the single molecule in the other two *Broussonetia* mitogenomes. A similar pattern was detected in okra (*Abelmoschus esculentus*) with circular and a liner-branching structure concurrently as well as *Silene vulgaris* with two independent molecules (Sloan et al., 2012b). In some cases, three or more multichromosomal circular mitogenomes could be observed like *Cucumis sativus* (Alverson et al., 2011) and *Silene conica* (Sloan, 2013). These observations suggest that multi-molecules may be more frequent than expected. Few genes shared between the two molecules indicated their necessary cooperation to function as normal. The polymorphisms in the conformation of the plant mitochondrial genome always puzzle us. Our results also supported the representation that plant mitogenomes should be considered a dynamic genome, at least, this structure is a more complete description of complicated plant mitogenomes (Li et al., 2022). The most complicated structure was observed in the *B. papyrifera* mitogenome because it has six repeats that could mediate 19 kinds of connections, but the *B. monoica* mitogenome had two repeats controlling seven kinds of connections. All these connections indicated an alternative configuration. To perform further analyses, a conventional single molecular structure was resolved in *B. papyrifera* and *B. monoica* of 325,822 and 276,967 bp in size while a double-ring structure was resolved in *B. kaempferi* of 267,420 bp in total size with 151,895 bp in the larger ring (RA) and 115,525 bp in the smaller one (RB). The varying size and structure may in part be the result of a limited number of repetitive sequences, which are known to be involved with large structural rearrangements (Abdelnoor et al., 2003; Hofmann, 2011), as the relative larger mitogenome size of *B. papyrifera* was due to a pair of 33-kb repeats (**Figures 1**, **2**). During the evolutionary history, massive original mitochondrial genes have transferred into nuclear region for more stable inner environment or functional efficiency across different lineages (Wicke et al., 2011) and about 30 to 50 encoding genes remained. The three *Broussonetia* mitogenomes shared 36 unique genes including 23 PCGs, two rRNA genes, and 11 tRNA genes, and several tRNA genes were lost in *B. kaempferi* and/or *B. papyrifera* (**Table 1**, **Figure 2**), which was common in other species such as *Dalbergia odorifera (* Hong et al., 2021 *)* and *Abelmoschus esculentus* (Li et al., 2022). The conservation in gene content across many plant lineages indicated the stable function of the mitogenome as it is responsible for the basic energy synthesis for life activities through oxidative phosphorylation (Mackenzie and McIntosh, 1999). And obviously it is the evidence of “evolutionary paradox” of plant mitogenomes. The subsequent phylogenetic analysis based on organellar genes supported a closer relationship between *B. kaempferi* and *B. monoica* with *B. papyrifera* diverging the earliest within *Broussonetia* (**Figure 5**), and that was consistent with the synteny pattern wherein more bulks of synteny could be found between these two rather than with *B. papyrifera* indicating a more similar sequence component and arrangement (**Figure 3**).

RNA editing events are pretty frequent in plant mitogenomes [20]. RNA editing in functional genes can result in massive diversity in gene sequence beyond what is encoded at DNA level. These diversities may be influenced by indels and substitutions of specific nucleotides at the RNA level (Sloan and Wu, 2016; Wu et al., 2022). The number of RNA editing sites in land plant mitogenomes can vary dramatically from zero in *Marchantia polymorpha* to 2152 sites in *Selaginella moellendorffii* (Zhang et al., 2020b). Here in the three *Broussonetia* spp., massive heterogeneity in RNA editing site abundance, type, efficiency, and location could be detected among species and tissues. The *B. kaempferi* mitogenome contained the most abundant sites, especially in the root and old stem, and in *B. monoica* mitogenome, the stipule was the most site-rich tissue. The C>T was dominant of the type of no matter in any organellar genome, species, or tissue. In several tissues, more sites in cds tended to high-effectively editing (**Figure 6**). Such massive heterogeneity among tissues might reflect the differences in activities based on functional divergence. Additionally, RNA editing may change the biochemical nature of the protein products by influencing functional structures. RNA editing events in *Arabidopsis mitochondria* showed that 35% of the modifications of the codons altered the amino acids from hydrophilic to hydrophobic. Also, RNA editing may contribute to protein stability and quantity (Jiang et al., 2022). It should be noted that although the average coverage was not that deep due to the low expression levels or a small amount of RNA sequencing data, it still provided plenty of information to further understand the potential functional roles that RNA editing has in the mitogenome. Given that RNA editing sites were not thought to exist in algae until recently, it suggests more attention to investigate RNA editing in a greater species range and that RNA editing is probably more common in later‐diverged land plants (Wu et al., 2022).

IGT as an intercellular type of horizontal gene transfer (HGT), widely exists in the plant genomes, occurs between organellar and nuclear genomes, and the DNA fragments from organellar genomes can be integrated into nuclear genomes to become NUMTs and NUPTs (Wang et al., 2012). Between the two plant organellar genomes, the most frequent direction is from plastome to mitogenome. Massive mitochondrial plastid fragments (MTPTs) could be detected in plant mitogenomes. In general, plant mitogenomes have about 0.56% (*Marchantia polymorpha*) – 10.85% (*Phoenix dactylifera*) plastid-derived sequences (Zhao et al., 2019). Here, we detected MTPTs in the three *Broussonetia* spp. and found that *B. monoica*, *B. kaempferi*, and *B. papyrifera* integrated 24 (30,604 bp), 27 (21,545 bp), and 26 (26,037 bp) MTPTs, respectively. They did not show great heterogeneity in MTPTs length distribution, and although *B. papyrifera* possessed the largest mitogenome size, its MTPTs were not the longest in length. These results indicate that the plastid-derived integrations could limitedly contribute to the mitogenome expansion in size. However, more analyses based on wide-ranged *Broussonetia* spp. are necessary to draw a precise conclusion. In addition, the integration of transferred fragments could be linked to DNA double-strand breaks (DSB) repair mechanisms such as homologous recombination (HR) and nonhomologous end joining (NHEJ), wherein several elements including transposable elements (TE), microhomology, and tRNA actively function (Zhao et al., 2019). Among the length of top-ranked regions, many tRNA genes could be detected, indicating they might take part in the integration of MTPTs.

## Conclusions

Although the plant mitogenome has unresolved characteristics, it obviously has important functions for evolution and life activities. In this paper, we assembled three *Broussonetia* high-quality mitogenomes and performed a comprehensive comparison in terms of structure, gene content, codon usage, and IGTs to provide a detailed genome landscape. Single or double circular forms showed intrageneric complexity of configuration. Despite gene content being quite conserved the numbers of copies changed, especially in some tRNAs. IGTs tended to transfer from certain regions of plastomes but integrated randomly flanking with tRNAs across the mitogenomes. Additionally, the abundance of RNA editing sites was uneven in different species and tissues, but C>T sites were more frequent in general, and most RNA editing happened in CDS, together with a relatively higher editing efficiency. Intergenic nuclear mitochondrial DNA segments were transcribed at various levels, most of which were ORFs. Our analysis here will promote the understanding of plant mitogenome structure and evolution, which will benefit plant mitogenome evolutionary research in the future.

## Materials and methods

### Plant material and sequencing

Three *Broussonetia* spp. examined in this study were collected from Zhejiang Province, China (*B. monoica*, 28°43’ N, 120°35’ E; *B. kaempferi*, 27°54’ N, 120°43’E; *B. papyrifera*: 27°55’N, 120°41’ E). The total DNA was extracted from fresh young leaves following the cetyltrimethylammonium bromide (CTAB) method (Doyle and Doyle, 1987). Illumina pair-end (PE) reads was generated by the Illumina NovaSeq platform (Illumina, San Diego, CA, USA) and long reads (ONT) was generated using a PromethION sequencer (Oxford Nanopore Technologies, Oxford, UK). Tissue-specific RNA sequencing libraries were generated using NEBNext^®^ Ultra™ RNA Library Prep Kit for Illumina^®^ (#E7530L, NEB, USA) following the manufacturer’s recommendations and were sequenced on an Illumina platform by Wuhan Benagen Technology Co., Ltd. to generate 150 bp paired-end reads.

### Mitogenome assembly and annotation

Given the abundance of plastome and mitogenome within a cell, the clean Illumina PE reads of whole-genome sequencing were first randomly extracted using SeqKit 0.13.1 (Shen et al., 2016) to generate about 6 Gb and 3 Gb datasets for mitogenome and plastome assembly, respectively. A combining strategy was performed to obtain accurate mitogenomes. The extracted PE reads were *de novo* assembled with five independent processes in SPAdes 3.15.2 (Bankevich et al., 2012) wherein K-mer was set at five values (51, 71, 91, 101, and 121) and further combined to obtain scaffolds. The coding sequence (CDS) of *Hemiptelea davidii* mitogenome (MN061667.1) was used as the reference to align against the scaffolds for excluding non-mitogenomic fragments using Bandage 0.8.1 (Wick et al., 2015). Mitogenomic sequences from Illumina were finally obtained after removing the fragments with abnormal depths compared to general mitochondrial sequences (10 and more times lower obviously from nuclear genome and over 100 times higher from plastome). The mitochondrial sequences were used to select ONT reads using BLAST 2.11.0+ (Ye et al., 2006) with 80% identity, before which ONT reads were self-corrected using Nextdenovo 2.3.1 (https://github.com/Nextomics/NextDenovo). Then the corrected ONT reads were *de novo* assembled using Flye 2.8.3 (Kolmogorov et al., 2019) followed by a three-round polish by PE reads using Pilon 1.23 (Walker et al., 2014). The final mitogenomes were generated after adjustment using Bandage 0.8.1. To confirm the assembly accuracy, polymerase chain reaction (PCR) was performed to verify connections intermediated by short repeats. The reaction mix contained 7 μL ddH_2_O, 1 μL upstream primer, 1 μL downstream primer, 1 μL cDNA template, and 10 μL 2×Lightning Taq PCR Master Mix. The settings for PCR were as follows: 94°C for 5 min, 94°C for 1 min, 60°C for 1 min, 72°C for 1 min, with 30 cycles. The primers were designed using Primer Premier 6.0 (Singh et al., 1998) and provided in **Table S1**. Illumina reads were also mapped against the final assembly. For the plastome assembly, extracted PE reads were aligned against three published congeneric plastomes (NC_035569.1, NC_047183.1, and MH223642.1) to filter out the plastomic reads using BWA-MEM algorithm in bwa 0.7.17 (Li and Durbin, 2009) and then used for *de novo* assembly in SPAdes 3.15.2 with five k-mers as above. Bandage 0.8.1 were used to obtain a circular molecule. Both PE and ONT reads were mapping back against the final assemblies and sliding windows (bin = 500, and step size = 200) were performed to calculate sequencing depth across mitogenomes using Bedtools 2.30.0 (Quinlan and Hall, 2010).

To obtain accurate annotations for the six organellar genomes, Geseq (https://chlorobox.mpimp-golm.mpg.de/geseq.html) (Tillich et al., 2017) was used with *Morus notabilis* (NC_041177.1) as reference for mitogenome, and three *Broussonetia* spp. (NC_035569.1, NC_047183.1, and MH223642.1) for plastome. All annotations were further manually verified and corrected. The genome map was generated using OGDRAW 1.3.1, (https://chlorobox.mpimp-golm.mpg.de/OGDraw.html) (Greiner et al., 2019). The final assembly and annotation files were submitted to NCBI (https://www.ncbi.nlm.nih.gov/).

### Repeats detection and codon usage analysis

Dispersed repeats were detected using the REPuter online program (https://bibiserv.cebitec.uni-bielefeld.de/reputer) with the following settings: hamming distance = 3, minimal repeat size = 30 (90% sequence identity or greater), maximum computed repeats = 5,000, and an e-value cutoff = 1e-5 (Kurtz et al., 2001). Simple sequence repeats (SSRs) were detected using MISA (Beier et al., 2017) including motif sizes from one to six nucleotide units with repeat lower thresholds set to of 8, 5, 4, 3, 3, and 3 repeat units for mono-, di-, tri-, tetra-, penta-, and hexa-nucleotide SSRs, respectively. REPuter (Kurtz et al., 2001) was applied to detect long repeats across the chloroplast genome with default settings. The relative synonymous codon usage (RSCU) of the mitogenome was calculated using DAMBE 5.2.73 (Xia and Xie, 2001).

### Synteny and transfer fragment analysis

MAFFT 7 (Katoh et al., 2017) was used to initially compare the organellar genomes of each species. To further detect the transferred fragments between plastome and mitogenome, BLAST 2.11.0+ was used to search for the homologous fragments with e-value 1e-5 and 80% identity as cutoff as previously described (Hong et al., 2021). Fragments shorter than 100 bp were excluded. To eliminate redundant detections, only single IR of each plastome was used for analysis, and fragments with overlapping positions in either plastome or mitogenome were merged to be unique. Bedtools 2.30.0 was used to split mitogenomes and plastomes into 5000-bp and 3000-bp windows, respectively, in each the total length of transferred fragment was calculated to demonstrate the hotpot. Interspecies homogonous regions were searched for to indicate the mitogenomic synteny among three *Broussonetia* species, fragments shorter than 100 bp were excluded from the analysis. The synteny was visualized using Circos 0.69 (Krzywinski et al., 2009).

### Organellar phylogenetical inference

Considering all the available mitogenomic data in NCBI, mitogenome and plastome of six species were selected from GenBank for use in the organellar phylogenetic analysis, including *Allaeanthus kurzii*, (MH311530.1, NC_041637.1), *B. monoica*, MH311528.1, NC_047181.1), two *Morus* spp. (*M. notabilis*, NC_041177.1, NC_027110.1; *M. alba*, MW924383.1, NC_057087.1), *Trophis scandens* (MH311529.1, MH189568.1), and *Hemiptelea davidii* (MN061667.1, NC_063957.1) as the outgroup. All shared CDS were aligned in MEGA 7 (Kumar et al., 2016). Those well-aligned CDS (13 for mitogenome and 73 for plastome) were concatenated by Phylosuit (Zhang et al., 2020a) to generate two organellar matrices after passing incongruence length difference (ILD) in PAUP 4.0 (Swofford, 2002) and substitution saturation test in DAMBE 5.2 (Xia et al., 2003). DnaSP 6 (Rozas et al., 2017) was used to calculate different types of matric sites. The maximum likelihood (ML) analyses were implemented in IQ-Tree 2.1 (Nguyen et al., 2015) with 1,000 bootstrap (BS) replicates to assess clade support. The optimal model was determined through ModelFinder 1.6.8 (Kalyaanamoorthy et al., 2017) using the corrected Akaike Information Criterion (AICc). The final trees were visualized in Figtree 1.4 (http://tree.bio.ed.ac.uk/software/figtree/).

### RNA-seq data analysis

RNA-seq data were firstly filtered using trimmomatic 0.39 (Bolger et al., 2014) to obtain high-quality clean reads (settings: MINLEN:50 LEADING:20 TRAILING:20 SLIDINGWINDOW:5:20). HISAT2 2.2.1 (Pertea et al., 2016) was used for the alignment of RNA reads from different tissues to our assembled mitogenomes. To detect the frequent RNA editing sites (C>T and T>C) (Gray, 2009) in organellar genomes, Samtools 1.7 (https://www.sciencedirect.com/topics/neuroscience/samtools) and Bedtools 2.30.0 were used to call SNPs and process VCF files, respectively. Given the abundance of mitogenome and plastome, SNPs with depth < 10 and 20, respectively, or quality < 10 were removed from the datset, SNPs located within 5 bp near indel were also excluded. To evaluate the transcript abundance of mitogenomic transfer to nuclear regions inferred from overall low mitogenomic transcripts, counts of RNA mapping reads generated by HiSAT2 2.2.1 were calculated through a dedicated sliding window (bin = 100 bp, and step size = 50) using Bedtools 2.30.0. Intergenic space with relatively high transcription was verified using BLASTn (https://blast.ncbi.nlm.nih.gov/) and Open Reading Frame (ORF) Finder (https://www.ncbi.nlm.nih.gov/orffinder/) (Rombel et al., 2002).

## Data availability statement

The data presented in the study are deposited in the GenBank repository, accession number OP342744, OP342745, OP342746, and OP342747.

## Author contributions

YZ, ZW, JW and CL conceived and designed the study. CL, JW, and SZ performed the experiments and data analysis. PL, CL and YZ contributed materials collection. JW, and SK wrote the paper. ZW, PL, WR, and YZ revised the paper. All authors contributed to the article and approved the submitted version.

## Funding

This study was supported by the Research Funds for the Natural Science Foundation of Zhejiang Province (Grant No. LY21C030002) and the Scientific Research Project of Baishanzu National Park (Grant No. 2021KFLY06) to YZ. And it also co-funded by the National Natural Science Foundation of China (31970244 and 32170238), and the Science, Technology, and Innovation Commission of Shenzhen Municipality (RCYX20200714114538196) to ZW.

## Conflict of interest

The authors declare that the research was conducted in the absence of any commercial or financial relationships that could be construed as a potential conflict of interest.

## Publisher’s note

All claims expressed in this article are solely those of the authors and do not necessarily represent those of their affiliated organizations, or those of the publisher, the editors and the reviewers. Any product that may be evaluated in this article, or claim that may be made by its manufacturer, is not guaranteed or endorsed by the publisher.
