# Supplementary material for: Comparative analysis of mitochondrial genomes of Broussonetia spp. (Moraceae) reveals heterogeneity in structure, synteny, intercellular gene transfer, and RNA editing
Source: Front Plant Sci. 2022 Dec 1;13:1052151. doi: 10.3389/fpls.2022.1052151 (PMC9751378; doi:10.3389/fpls.2022.1052151)
Supplement: Supplementary file 1 [file DataSheet_1.docx]

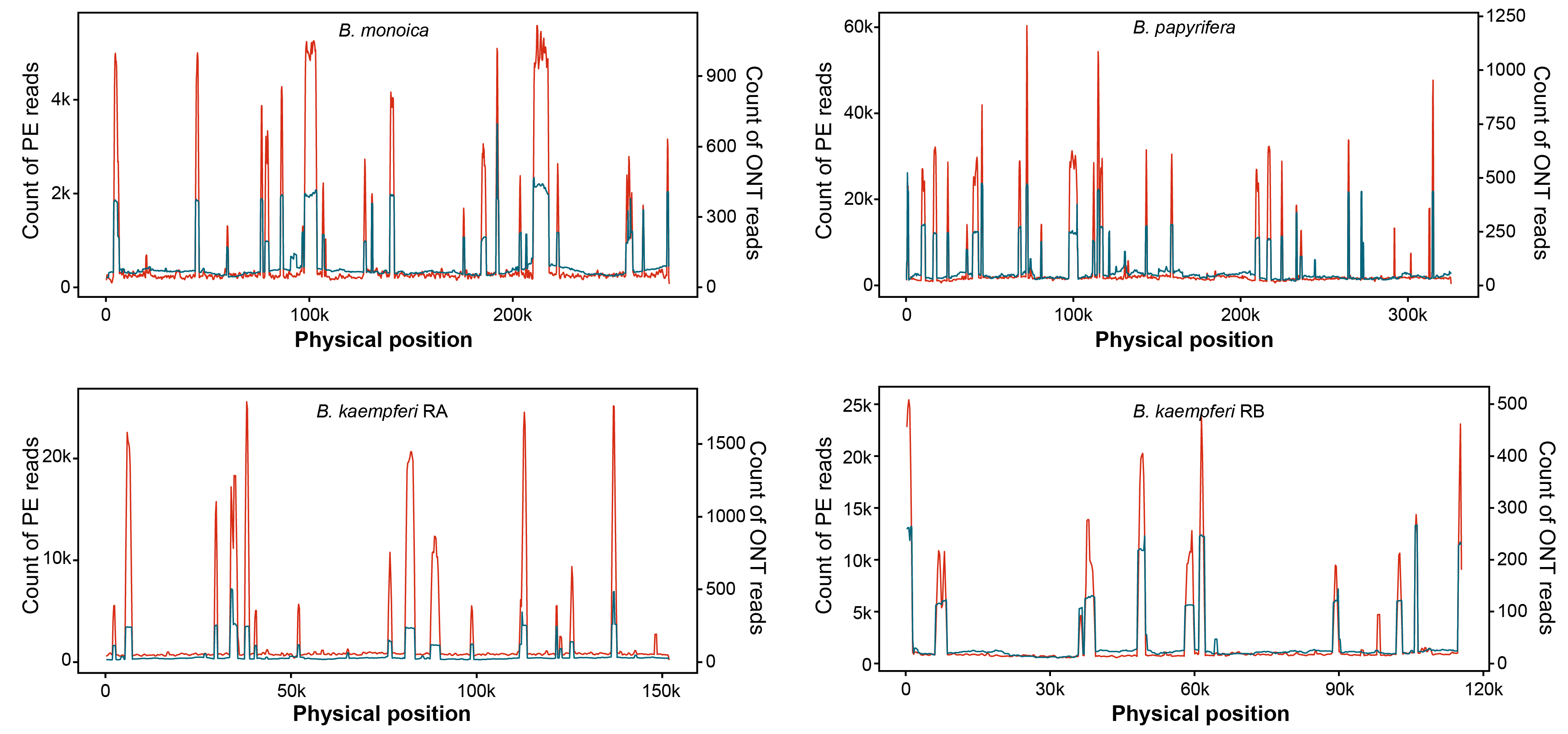


Figure S1. Sequencing depth (count of mapping reads) of *Broussonetia* mitogenomes in this study. Counts of PE and ONT reads are indicated by red and blue lines, respectively.


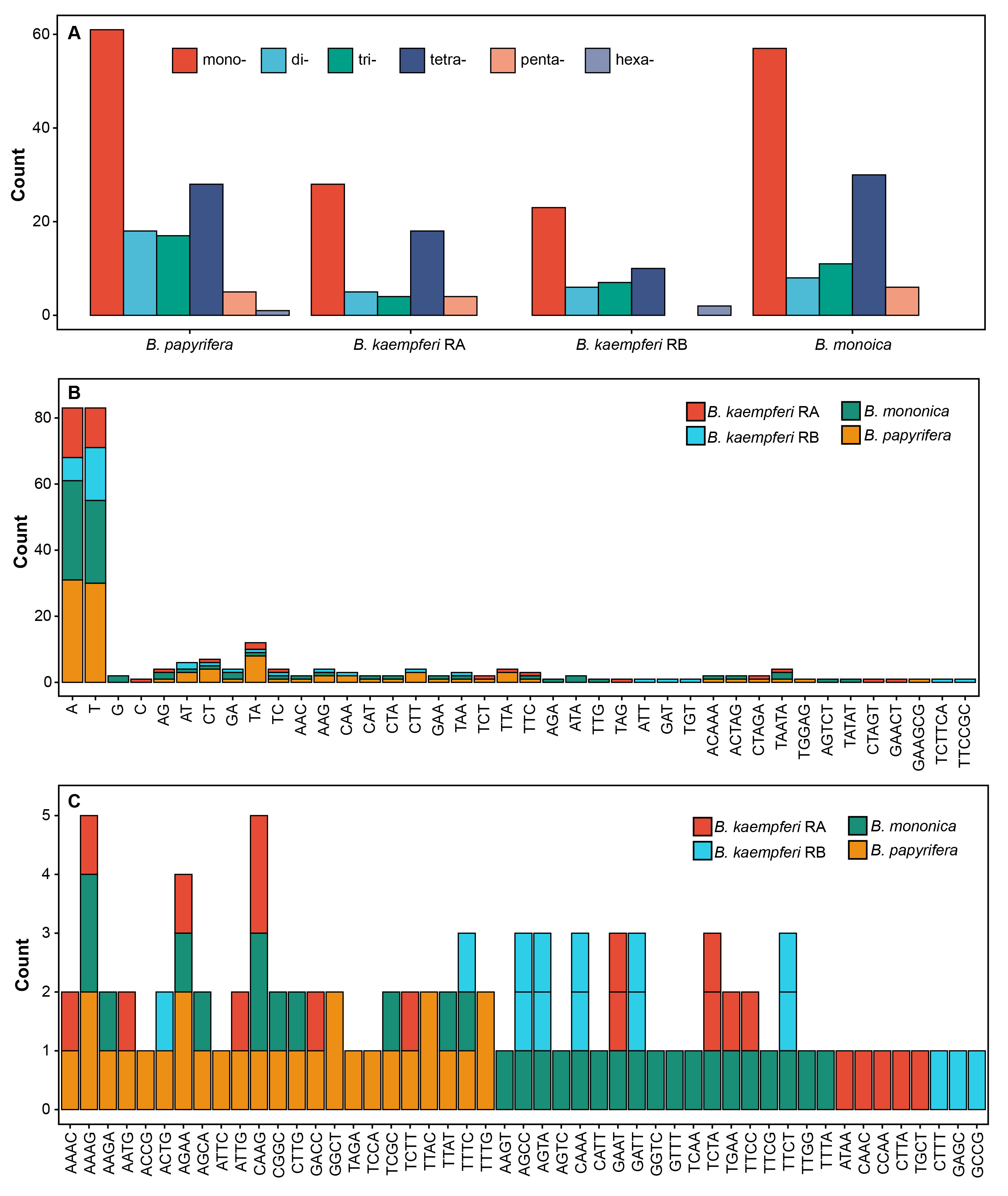


Figure S2. SSRs in three *Broussonetia* spp. mitogenomes. (A) Count of six unit-sized SSRs. (B) Count of different unit components from mono- to hexa- except for tetra-nucleotide SSRs. (C) Count of different unit components of tetra-nucleotide SSRs.


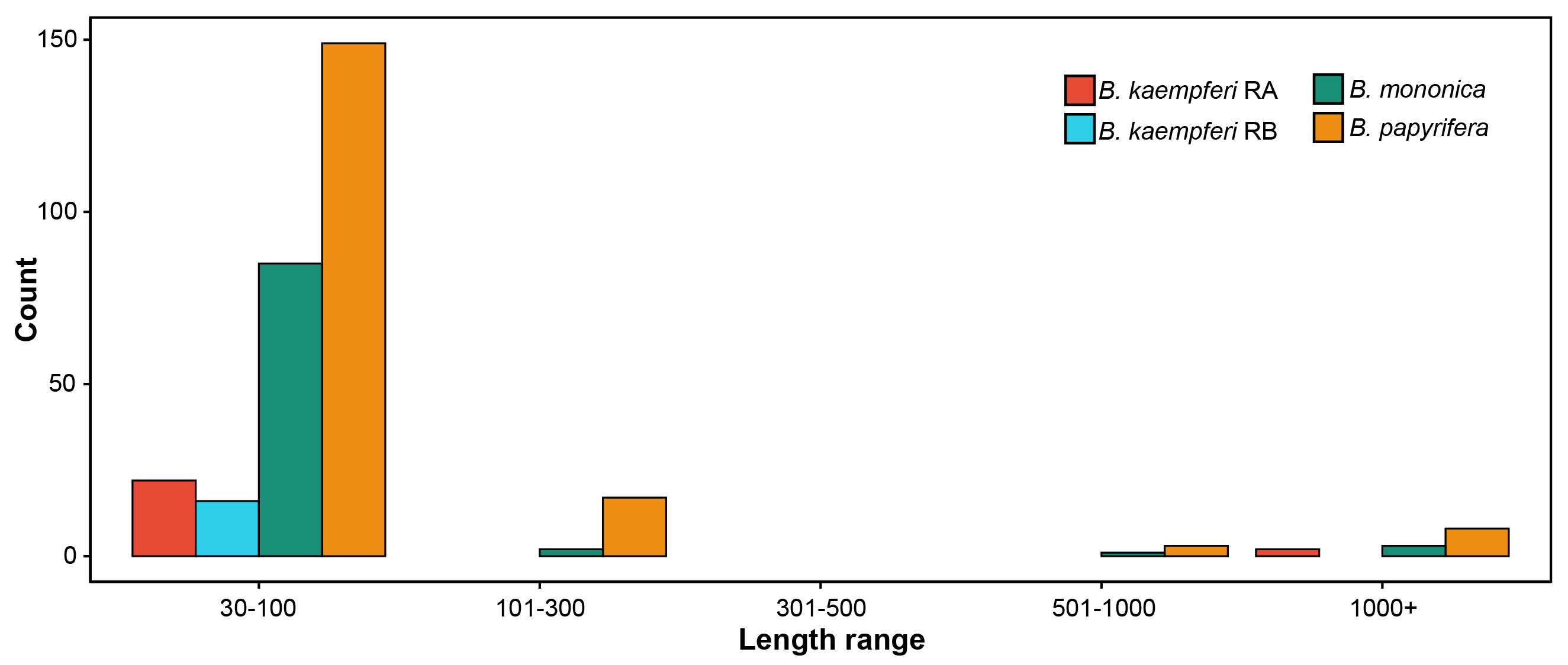


Figure S3. Length of dispersed repeats in three *Broussonetia* spp. mitogenomes.





Figure S4. Interspecific mitogenomic synteny indicated by homologous regions shorter than 5000 bp.


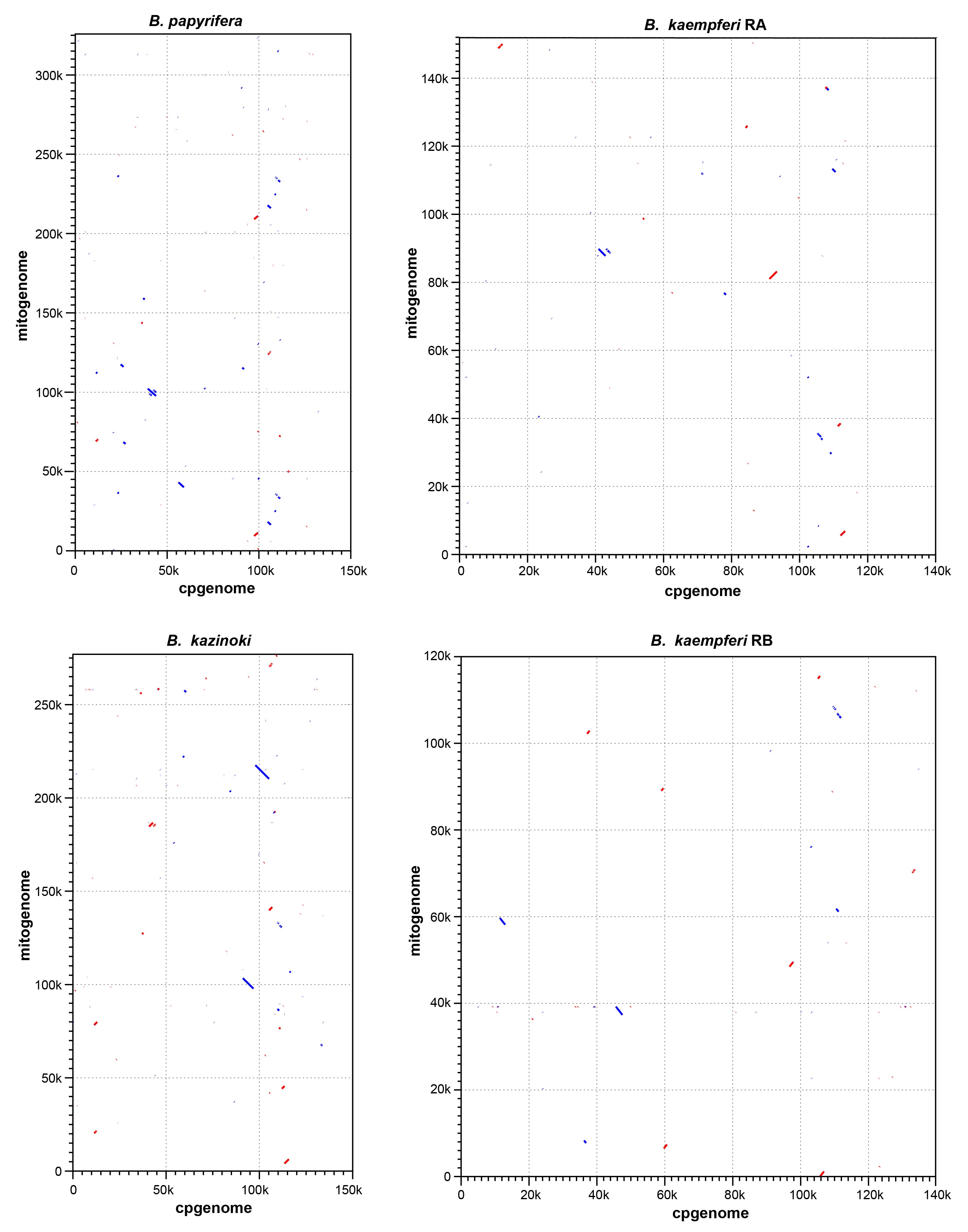


Figure S5. Dot-plot of alignment between organellar genomes of each species. Forward and reverse alignments are indicated by red and blue dots, respectively.


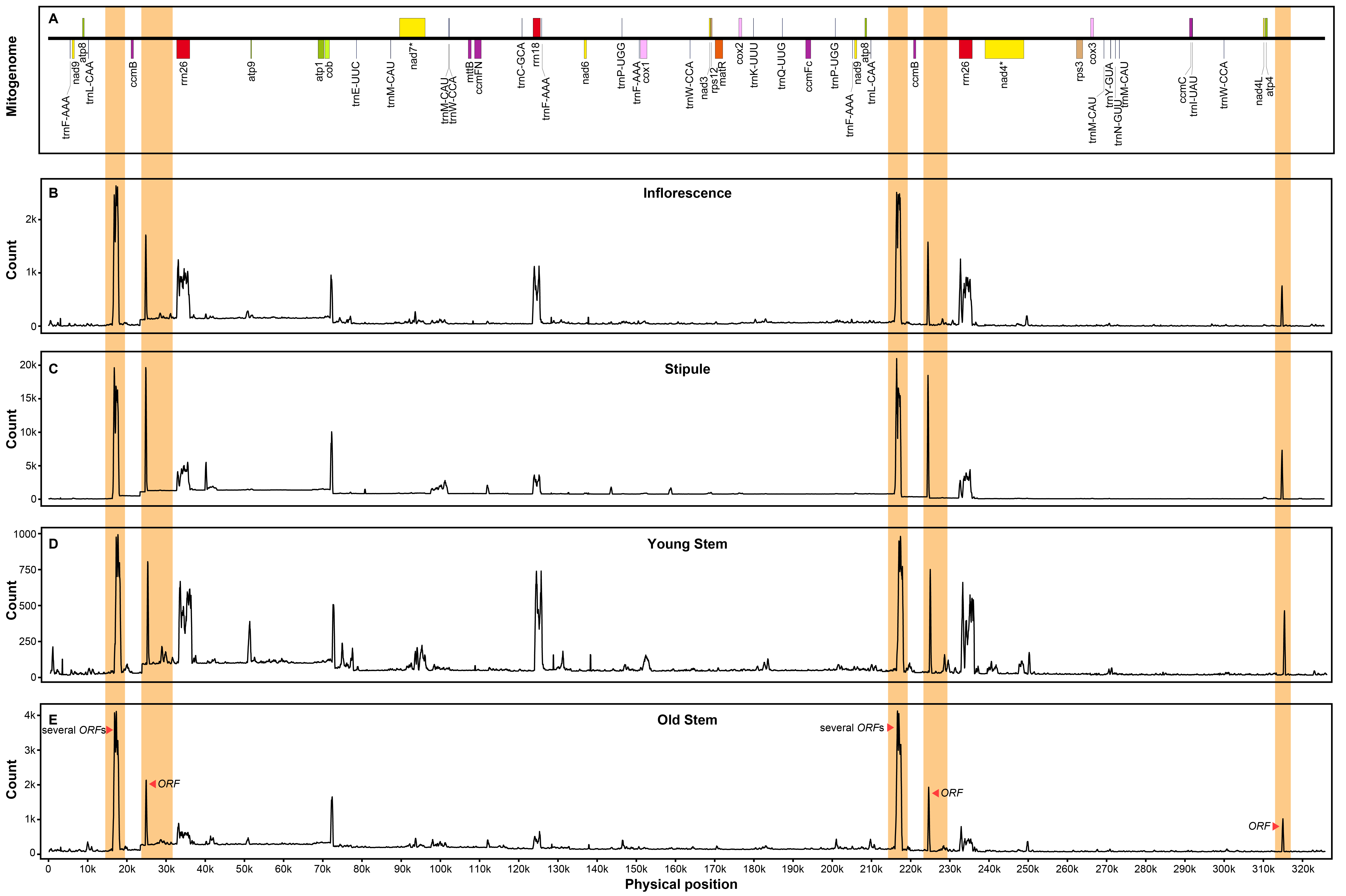


Figure S6. Transcript abundance across *B.* *papyrifera* mitogenome transfer into nuclear genome in different tissues. Orange regions indicates five IGS (two IGS with two copies for each) with relatively high transcript abundance. (A) Liner annotation of *B. papyrifera* mitogenome. (B-E) Count of RNA reads mapping to *B. papyrifera* mitogenome in inflorescence, stipule, young stem, and old stem, respectively, using a 100-bp sliding window with a 50-bp step size. Red triangles indicate annotated ORFs.


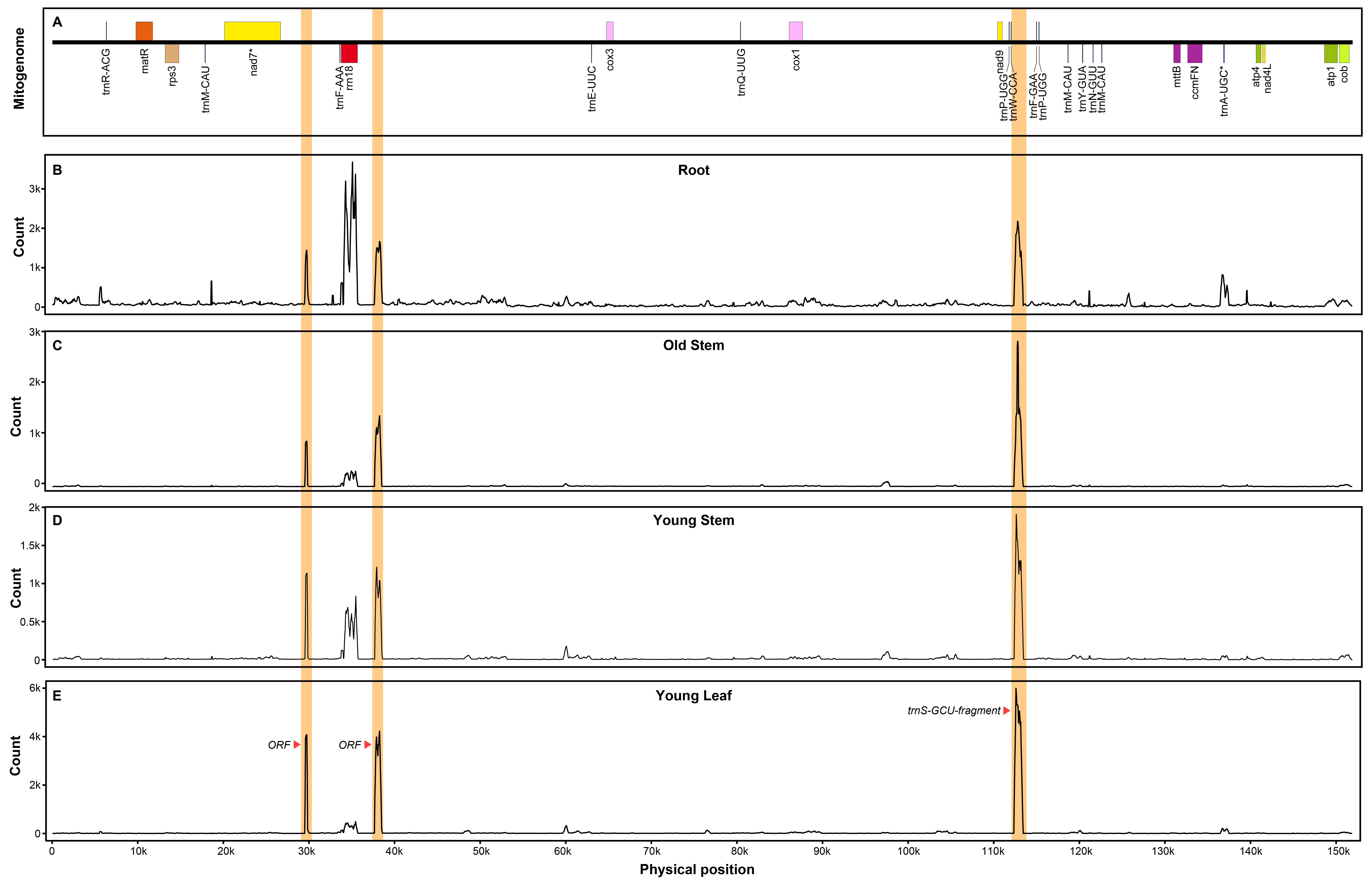


Figure S7. Transcript abundance across *B. kaempferi* RA mitogenome transfer into nuclear genome in different tissues. Orange regions indicates five IGS (two IGS with two copies for each) with relatively high transcript abundance. (A) Liner annotation of *B. kaempferi* RA mitogenome. (B-E) Count of RNA reads mapping to *B. kaempferi* RA mitogenome in root, old stem, young stem, and young leaf, respectively, using a 100-bp sliding window with a 50-bp step size. Red triangles indicate annotated gene fragments and ORFs.


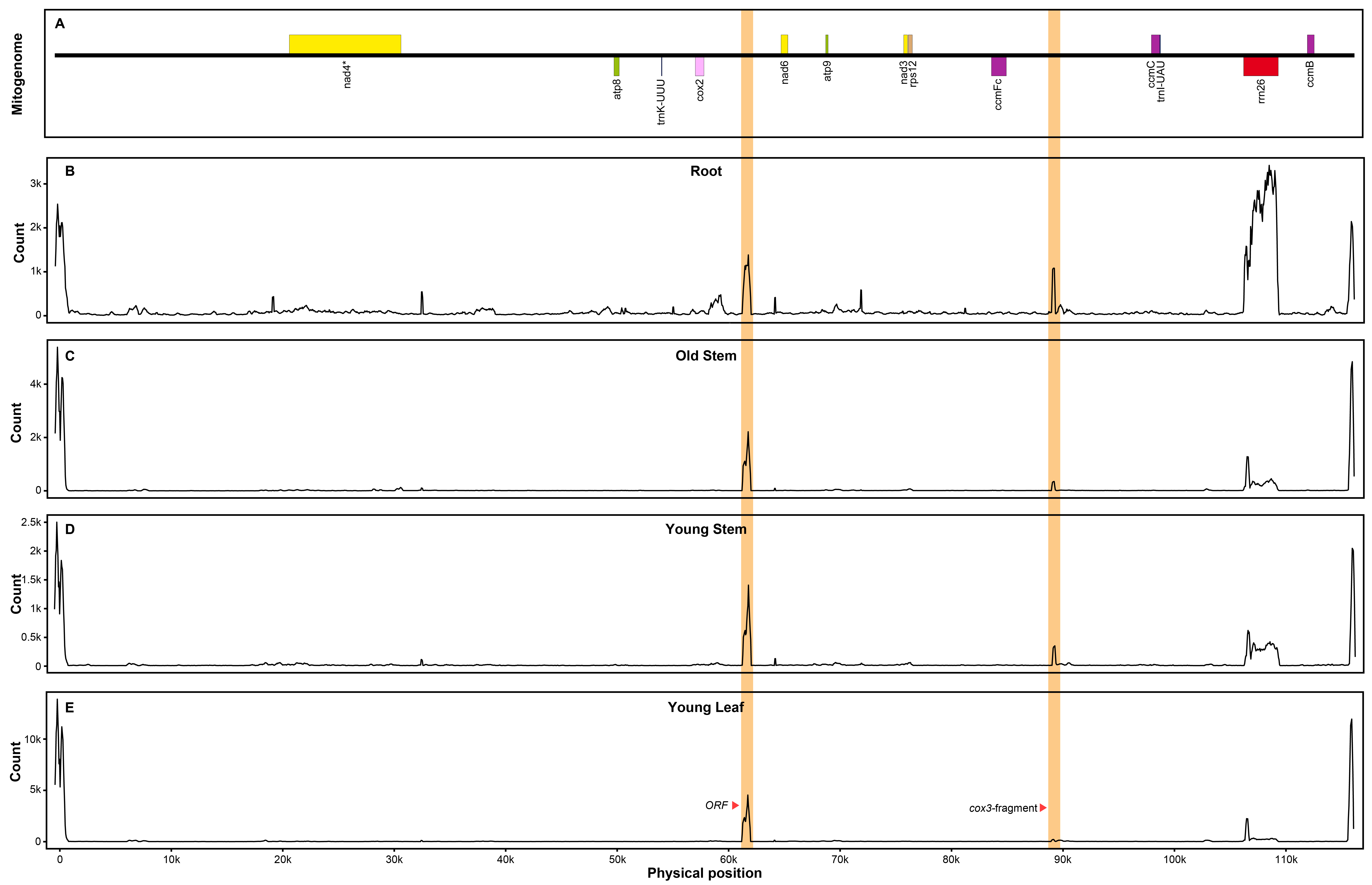


Figure S8. Transcript abundance across *B. kaempferi* RB mitogenome transfer into nuclear genome in different tissues. Orange regions indicates five IGS (two IGS with two copies for each) with relatively high transcript abundance. (A) Liner annotation of *B. kaempferi* RB mitogenome. (B-E) Count of RNA reads mapping to *B. kaempferi* RB mitogenome in root, old stem, young stem, and young leaf, respectively, using a 100-bp sliding window with a 50-bp step size. Red triangles indicate annotated gene fragments and ORFs.

Table S1. Primer pairs used for verification of mitogenomic connections mediated by repeats.

| **No.** | **Species** | **Connection** | **Junction** | **Upstream primer** | **Downstream primer** | **Product length** |
| --- | --- | --- | --- | --- | --- | --- |
| 1 | *B. monoica* | edge_6-edge_1 | 59,359 | CAAGCAAGCAAGCCATCCAAGT | TCAGTCAGCGTCACTCCTTCCT | 581 |
| 2 | *B. monoica* | edge_5-edge_1 | 88,423 | AACCGCCGCTTGCTCCTATTC | CTTGGATGGCTTGCTTGCTTGT | 927 |
| 3 | *B. monoica* | edge_3-edge_2 | 7,301 | GCCCGACTCCAAGTTGTTCAAG | GTCTTTGTGCGAGCCGTATGC | 1,100 |
| 4 | *B. monoica* | edge_1-edge_2 | 1,589 | AGGAAGGAGTGACGCTGACTGA | GGCGAGATCCAACGGTGAACA | 277 |
| 5 | *B. monoica* | edge_2-edge_5 | 883 | AGCGTGCGTTGCGTGATTCT | TTGCCGTGCCGTGAAGTGAA | 934 |
| 6 | *B. monoica* | edge_2-edge_4 | 883 | TACCACCCACCCTACCCTACCA | TAGCGGCAGCAACAGCGATAAG | 1,242 |
| 7 | *B. monoica* | edge_2-edge_4’ | 883 | AGCGTGCGTTGCGTGATTCT | TTGCCGTGCCGTGAAGTGAA | 934 |
| 8 | *B. papyrifera* | edge_11-edge_1 | 32,462 | ACGAACCAGCGGAGGAACATCT | AACAACTCGGCACTCGCATCG | 1,700 |
| 9 | *B. papyrifera* | edge_1-edge_13 | 740 | TCGGCACTCGCATCGGTGAA | ACGCTGGATGGAACGCCCTTA | 791 |
| 10 | *B. papyrifera* | edge_2-edge_1 | 48,937 | AGGCTGTAAGGCAGAGCGAATC | AAGCTGGAGCTGAGCGAGGAT | 795 |
| 11 | *B. papyrifera* | edge_1-edge_3 | 740 | CGATGCGAGTGCCGAGTTGT | GGTGTGCCTTTGGGAACTGCTA | 541 |
| 12 | *B. papyrifera* | edge_1-edge_4 | 740 | CGATGCGAGTGCCGAGTTGT | GGCTGCTCAAGGCTATGTTCCT | 669 |
| 13 | *B. papyrifera* | edge_8-edge_4 | 11,911 | GCAAGACACTCGCTTCCACAAC | GTCAACAACGCTTCGCACAACT | 1,425 |
| 14 | *B. papyrifera* | edge_4-edge_9 | 5,503 | GCCACGTCACCTAGAAGTCTGT | TTGCCGTAACTGCTCAGGATGT | 1,657 |
| 15 | *B. papyrifera* | edge_2-edge_5 | 48,936 | TTCCGAACGAACCAGCAGCAA | CGTGAGTCCTCGCCGTAACATT | 1,753 |
| 16 | *B. papyrifera* | edge_3-edge_5 | 2,466 | GCCGTTGAGAGCGTCTGTTGTC | ACCTCCAGCCAATGCGTTGAGA | 1,622 |
| 17 | *B. papyrifera* | edge_5-edge_6 | 2,731 | CCTGAAGCACGAACGGTAGTCT | CCAACCTCCACCAGCAAGCA | 1,275 |
| 18 | *B. papyrifera* | edge_5-edge_7 | 2,731 | CGTATTCGTCCATAGGTCAA | CCACAAGCGGTAAGAGTC | 1,775 |
| 19 | *B. papyrifera* | edge_6-edge_8 | 1,442 | GCCATCCTTGAGTTCGCTGCTA | GCTTGCTCGCCTTCCTTCCA | 1,201 |
| 20 | *B. papyrifera* | edge_6-edge_10 | 1,442 | CATCGCTGTCTTGGCTGTGCTA | ACATCCATACCTTCCGCAGACC | 602 |
| 21 | *B. papyrifera* | edge_10-edge_11 | 33,902 | ACTCCGCTTCGCTACGTTCCTT | GGTGTGCTCGTTCCGCTTCATC | 1,279 |
| 22 | *B. papyrifera* | edge_12-edge_11 | 58,683 | TATCGCCACGCCGACCATCAA | GGTGTGCTCGTTCCGCTTCATC | 1,945 |
| 23 | *B. papyrifera* | edge_12-edge_14 | 58,683 | GAGGCTTGGCTGTACTGGATGA | TGGCAACGAACACTGACTTGGT | 2,068 |
| 24 | *B. papyrifera* | edge_13-edge_14 | 9,426 | ACAGACAGGAGCGAGCGAGAT | TGGCAACGAACACTGACTTGGT | 1,596 |
| 25 | *B. papyrifera* | edge_7-edge_14 | 24,378 | TGGCTCGTCCGAATTGTTCTGA | CGTAGTGAAGGTTGCTGTGAGG | 1,375 |
| 26 | *B. papyrifera* | edge_9-edge_14 | 34,142 | AGCACGAGACGGCGGTCAAT | GGAAGAGGCACGATAGCGAAGA | 1,127 |

Table S2. RSCU of three *Broussonetia* spp. mitogenomes. Codons with RSCU > 1 are bold and underlined.

| Amino acid | Codon | RSCU | | | Amino acid | Codon | RSCU | | | Amino acid | Codon | RSCU | | |
| --- | --- | --- | --- | --- | --- | --- | --- | --- | --- | --- | --- | --- | --- | --- |
|  |  | *B. monoica* | *B. kaempferi* | *B. papyrifera* |  |  | *B. monoica* | *B. kaempferi* | *B. papyrifera* |  |  | *B. monoica* | *B. kaempferi* | *B. papyrifera* |
| Stop codon | UAA | 0.952 | 0.986 | 0.958 | Ile | AUC | 1.034 | 0.966 | 0.973 | Arg | CGC | 0.913 | 0.878 | 0.847 |
|  | UAG | 1.006 | 0.991 | 1.011 |  | AUU | 0.991 | 1.038 | 1.037 |  | **CGG** | 1.077 | 1.128 | 1.092 |
|  | **UGA** | 1.042 | 1.023 | 1.031 | Lys | **AAA** | 1.042 | 1.026 | 1.055 |  | CGU | 0.818 | 0.792 | 0.794 |
| Ala | GCA | 0.988 | 1.062 | 0.970 |  | AAG | 0.958 | 0.974 | 0.945 | Ser | **AGC** | 1.049 | 1.050 | 1.059 |
|  | GCC | 1.013 | 0.966 | 0.977 | Leu | CUA | 0.903 | 0.955 | 0.913 |  | AGU | 0.951 | 0.950 | 0.941 |
|  | GCG | 0.703 | 0.694 | 0.710 |  | CUC | 0.999 | 0.942 | 0.939 |  | UCA | 0.974 | 0.984 | 0.968 |
|  | **GCU** | 1.295 | 1.278 | 1.344 |  | CUG | 0.719 | 0.721 | 0.720 |  | **UCC** | 1.032 | 1.085 | 1.047 |
| Cys | UGC | 1.014 | 0.957 | 1.032 |  | **CUU** | 1.379 | 1.381 | 1.429 |  | UCG | 0.791 | 0.703 | 0.754 |
|  | UGU | 0.986 | 1.043 | 0.968 |  | UUA | 0.949 | 0.928 | 0.933 |  | **UCU** | 1.203 | 1.228 | 1.231 |
| Asp | GAC | 0.828 | 0.808 | 0.840 |  | **UUG** | 1.051 | 1.072 | 1.067 | Thr | ACA | 1.012 | 0.989 | 0.943 |
|  | **GAU** | 1.172 | 1.192 | 1.160 | Met | AUG | 1.000 | 1.000 | 1.000 |  | **ACC** | 1.039 | 1.127 | 1.096 |
| Glu | **GAA** | 1.170 | 1.207 | 1.171 | Asn | AAC | 0.899 | 0.914 | 0.901 |  | ACG | 0.769 | 0.705 | 0.756 |
|  | GAG | 0.830 | 0.793 | 0.829 |  | **AAU** | 1.101 | 1.086 | 1.099 |  | **ACU** | 1.179 | 1.179 | 1.206 |
| Phe | UUC | 1.003 | 1.016 | 0.961 | Pro | **CCA** | 1.032 | 1.027 | 1.009 | Val | GUA | 1.001 | 1.008 | 0.995 |
|  | UUU | 0.997 | 0.984 | 1.039 |  | **CCC** | 1.032 | 1.010 | 1.044 |  | GUC | 0.905 | 0.937 | 0.955 |
| Gly | **GGA** | 1.207 | 1.191 | 1.202 |  | CCG | 0.773 | 0.792 | 0.729 |  | GUG | 0.895 | 0.921 | 0.872 |
|  | GGC | 0.888 | 0.793 | 0.822 |  | **CCU** | 1.163 | 1.171 | 1.217 |  | **GUU** | 1.199 | 1.134 | 1.178 |
|  | **GGG** | 1.045 | 1.127 | 1.050 | Gln | **CAA** | 1.135 | 1.135 | 1.150 | Trp | UGG | 1.000 | 1.000 | 1.000 |
|  | GGU | 0.860 | 0.890 | 0.926 |  | CAG | 0.865 | 0.865 | 0.850 | Tyr | UAC | 0.852 | 0.880 | 0.872 |
| His | CAC | 0.909 | 0.883 | 0.897 | Arg | **AGA** | 1.121 | 1.113 | 1.103 |  | **UAU** | 1.148 | 1.120 | 1.128 |
|  | **CAU** | 1.091 | 1.117 | 1.103 |  | AGG | 0.879 | 0.887 | 0.897 |  |  |  |  |  |
| Ile | AUA | 0.974 | 0.996 | 0.990 |  | **CGA** | 1.192 | 1.202 | 1.267 |  |  |  |  |  |

Table S3. Single window containing 2k+ transfer fragments across plastome.

| Plastome | Start | End | Transfer length | Window content |
| --- | --- | --- | --- | --- |
| *B. monoica* | 30k | 33k | 3000 | partial *trnC-GCA-petN*, *petN*, *psbM*, *trnD-GUC*, partial *trnD-GUC-trnY-GUA* |
| *B. monoica* | 33k | 36k | 3000 | partial *trnD-GUC-trnY-GUA*, *trnY-GUA*, *trnE-UUC*, *trnT-GGU*, partial *psbD* |
| *B. monoica* | 39k | 42k | 3000 | partial *psbZ-trnG-GCC*, *trnG-GCC*, *trnM-CAU*, *rps14*, partial *psaB* |
| *B. papyrifera* | 39k | 42k | 2542 | partial *psbZ-trnG-GCC*, *trnG-Gcc*, *trnM-CAU*, *rps14*, partial *psaB* |
| *B. monoica* | 21k | 24k | 2445 | partial *rpoC2*, partial *rpoC1* |
| *B. monoica* | 27k | 30k | 2284 | partial *rpoB*, *trnC-GCA*, partial *trnC-GCA-petN* |
| *B. papyrifera* | 24k | 27k | 2251 | partial *rpoC1*, partial *rpoB* |
| *B. monoica* | 24k | 27k | 2249 | partial *rpoC1*, partial *rpoB* |
| *B. kaempferi* RA | 21k | 24k | 2129 | partial *rpoC2*, partial *rpoC1* |
| *B. papyrifera* | 57k | 60k | 2122 | partial *atpB*, partial *rbcL* |
| *B. papyrifera* | 42k | 45k | 2015 | partial *psaB*, *psaA*, partial *psaA-ycf3* |

Table S4. Single window containing 2k+ transfer fragments across mitogenome.

| Mitogenomes | Start | End | Transfer length | Window content |
| --- | --- | --- | --- | --- |
| *B. monoica* | 210k | 215k | 4796 | partial *trnY-GUA-trnV-GAC*, *trnV-GAC*, partial *trnV-GAC-trnL-CAA* |
| *B. monoica* | 100k | 105k | 3421 | partial *trnM-CAU-trnQ-UUG*, *trnQ-UUG*, partial *trnQ-UUG-ccmC* |
| *B. papyrifera* | 40k | 45k | 2943 | partial *rrn26-atp9* |
| *B. monoica* | 215k | 220k | 2542 | partial *trnV-GAC-trnL-CAA*, *trnL-CAA*, partial *trnL-CAA-atp8* |
| *B. monoica* | 95k | 100k | 2399 | partial *trnM-CAU-trnQ-UUG* |
| *B. papyrifera* | 95k | 100k | 2361 | partial *nad7-trnM-CAU* |
| *B. papyrifera* | 100k | 105k | 2196 | partial *nad7-trnM-CAU*, *trnM-CAU*, *trnW-CCA*, partial *trnW-CCA-mttB* |
| *B. monoica* | 75k | 80k | 2193 | partial *nad6-cox2* |
| *B. kaempferi* RA | 80k | 85k | 2156 | partial *cox3-trnQ-UUG*, *trnQ-UUG*, partial *trnQ-UUG-cox1* |
| *B. monoica* | 255k | 26k | 2104 | partial *nad4-trnW-CCA* |
| *B. kaempferi* RA | 85k | 90k | 2046 | partial *trnQ-UUG-cox1*, partial *cox1-nad9* |
| *B. kaempferi* RB | 35k | 40k | 2018 | partial *nad4-atp8* |

Table S5. Characterization of the sequence matrices used for organellar phylogenetic inference.

|  | Total number of sites | Sites with alignment gaps or missing data | Invariable sites | Variable sites | Singleton variable sites | | | Parsimony informative sites | |
| --- | --- | --- | --- | --- | --- | --- | --- | --- | --- |
|  |  |  |  |  | two variants | three variants | four variants | two variants | three variants |
| plastome | 54,143 | 1200 | 48,359 | 4584 | 3073 | 87 | 1 | 1285 | 138 |
| mitogenome | 13,251 | 388 | 12,587 | 276 | 196 | 1 | 0 | 75 | 4 |
